# Timely or detailed? The impact of information disclosure on citizen co-production

**DOI:** 10.3389/fpubh.2026.1774267

**Published:** 2026-06-01

**Authors:** Chunxin Zhang, Diyi Liu, Binbin Peng

**Affiliations:** 1 Tianjin University, Tianjin, China; 2 Nankai University, Tianjin, China

**Keywords:** co-production, detail, information disclosure, public health crisis, timeliness

## Abstract

During public health crisis, effective information disclosure is crucial for the government to guide citizens’ participation in social recovery. In this paper, we focus on the influence of the timeliness and detail of information disclosure on citizens’ co-production behavior. We divide citizens’ co-production behavior into online information concern behavior and offline pandemic compliance with preventive behavior. Adopting a mixed-methods design combining regression analysis and behavioral experiments, this study quantitatively analyzed more than 5,800 pieces of data from 293 prefecture-level cities and 158 citizens’ experimental data. The results indicate that timely and detailed information disclosure is associated with significant effects on citizens’ co-production behavior. However, due to constraints on government human resources and time, it is recommended to adopt a general content and timely disclosure policy for information concerning behavior. A detailed content but non-timely disclosure policy is recommended for citizen compliance with preventive behavior. Findings have important implications for governmental public health crisis management.

## 1 Introduction

Information disclosure is a paramount obligation of the government, with its fundamental purpose to enhance the transparency of government work (1). Disclosure of government information enables citizens to provide constructive feedback and make more effective decisions about resource allocation, ultimately promoting public welfare (2, 3). During public health crisis, governments are frequently required to provide more timely and accurate information to reduce panic and enhance citizen cooperation (4, 5). However, there is still a lack of detailed academic research on information disclosure during public health crisis, with most existing studies focusing on financial disclosure or the disclosure of daily government work (6). The few studies in this field focus on whether disclosure is made (7), but there is still a lack of research on the detailed forms of disclosure. Given the lack of government resources and time limits during public health crises, achieving timely and detailed disclosure can be challenging. Therefore, governments often need to choose between timeliness and level of detail when disclosing information. How information is disclosed with varying content and timing can have different effects on guiding citizens’ behaviors. It is crucial to explore ways for the government to effectively disclose information that promotes citizens’ engagement in pandemic preventive behaviors.

Co-production behavior of citizens is an important research topic in studying the joint response of citizens and the government to crises. This paper aims to investigate how information disclosure promotes citizens’ co-production behavior during public health crises. These crises highlight the need for governments to effectively, rapidly, and widely disseminate accurate and reliable information (1, 8, 9). Co-production is broadly defined as a variety of activities in which state actors and citizens collaborate to generate benefits (10, 11). During public health crisis, citizens’ co-production behavior refers to sharing information and actively cooperating with preventive measures (12). This behavior aims to enhance the quality of public services and improve crisis prevention and control. Notably, in situations where social service resources are limited and public health crisis have a wide-ranging impact, the successful implementation of various prevention measures is challenging without citizen cooperation (12, 13). Therefore, the co-production behavior of citizens has important research value. Current research on the impact of citizen co-production predominantly focuses on unified categories (14), and some studies categorizing individual co-production and collective co-production (14). During public health crises, social networks play a significant role in policy formulation (8, 15). Citizens can increase public understanding the crisis’s situation through online attention and dissemination of information. This helps to raise public awareness and participation, thereby having a positive impact on the entire society. Therefore, it can be regarded as a form of co-production behavior. At the same time, compliance with offline behavior is also a form of co-production behavior, because citizens’ compliance with measures can help reduce the risk of crises transmission and protect their health and safety. This behavior has important positive effects on the entire community, achieving the goal of co-production. Hence there are multiple types of citizens’ co-production behaviors, mainly comprising online and offline actions. These include citizens’ engagement in online activities such as following and sharing pandemic-related information, as well as offline behaviors like adhering to pandemic prevention measures and actively participating in volunteer services. However, there is currently insufficient research on the classification of co-production behavior and the extent to which online and offline behaviors are influenced by information disclosure. The degree to which co-production behaviors of different types are affected may not be the same. If co-production behaviors are studied according to a unified category, it may lead to incorrect analysis of the factors that affect them and result in erroneous guidance of citizen behavior. Targeted categorization studies can provide more effective foundations for government policy formulation. Citizens’ co-production behavior plays a crucial role in government crisis management, particularly during the outbreak of public health crisis (16). Hence, disclosing information during critical crisis can effectively promote government information dissemination and enhance citizen cooperation and support in emergency management (7). The COVID-19 pandemic provides a timely opportunity to study the effects of different forms of information disclosure during a crisis.

Given the research gap in the literature, this paper aim to make practical and academic contributions in the following three areas. Firstly, our study will focus on the impact of information disclosure on citizens’ different categories co-production behavior. Previous research on citizens’ co-production behavior has generally categorized it as a unified behavior where citizens and the government jointly produce benefits (11, 17). However, citizens’ co-production behavior during public health crisis can be classified into two types. One type involves concern and dissemination of government-disclosed information (18). The other type involves complying with preventive measures, such as wearing masks and regular disinfection (7). The reasons for these two types of behavior and the degree to which they are influenced by information disclosure may differ. For example, when the content of government information disclosure is general, people may fail to understand the severity of the pandemic and may be less willing to cooperate with preventive measures. Therefore, studying co-production behavior as a whole may provide ineffective guidance. Our study aims to examine these two categories of co-production behavior separately to provide better recommendations for guiding specific citizen behaviors. Secondly, this study focuses on the effects of different forms of information disclosure by applying signaling theory to advance the theoretical understanding of crisis communication. Currently, many governments engage in information disclosure, but new challenges have emerged regarding the specific operational aspects of disclosure. A simple “yes or no” answer regarding information disclosure does not provide sufficient guidance for the government; therefore, addressing these questions requires further research to explore whether different forms of information disclosure lead to varying impacts. When studying information disclosure forms, the most common aspects to consider are the level of detail in the disclosed content and the timeliness of disclosure (19, 20). Beyond the binary of disclosure, we conceptualize these dimensions as distinct institutional signals: specifically, we identify timeliness as a responsiveness signal reflecting governmental efficiency, and the level of detail as a competence signal reflecting administrative rigor. This framework allows us to uncover the inherent signaling trade-offs—speed versus granularity—and how these strategic choices influence citizens’ cognitive processing and subsequent participation during public health emergencies. In-depth research on different forms of information disclosure can provide valuable recommendations for governments to improve their information disclosure practices. This can help governments increase their credibility and promote citizens’ co-production behavior by providing them with detailed and actionable guidance. Thirdly, this study employs regression analysis and experimental methods for quantitative research. Previous co-production studies have mainly relied on qualitative methods (10, 21), and the measurement of citizens’ co-production has often relied on self-reported data (14, 22). Nevertheless, the research based on self-reported data may overstate the actual effect of the relationship. This study utilizes panel data and experimental data to investigate citizens’ co-production behavior during public health crisis, providing more reliable research findings.

## 2 Theoretical background

### 2.1 Citizens’ co-production

The term “co-production” was first introduced by Percy in 1978 (23). In the following decades, scholars further explored the role of citizens in designing, implementing, and overseeing public policies and services, recognizing the significance of citizen participation for public service delivery and social development (24, 25). Co-production has been defined in previous research as various activities where state actors and citizens work together to generate benefits (11). During public health crisis, there is an increased demand for citizen co-production in society. On one hand, a public health emergency encourages us to shoulder responsibilities collectively and maintain social stability (26). On the other hand, stimulating public self-reliance and encouraging citizens to provide voluntary assistance is essential for governments to allocate their limited resources and energy to support more vulnerable individuals (27). Co-production becomes more apparent than ever during public health crises, and it holds significant implications for government crisis management (14). Public health crisis requires temporary and intensive citizen involvement and compliance (12). Amid these situations, the social adherence to public health policies, such as maintaining social distancing and wearing masks, can be considered as a significant co-production endeavor (7).

However, citizens’ co-production behavior is not determined solely by individual will but is profoundly influenced by the structural determinants in which they are embedded. These factors are defined as the systemic roots of health inequality and injustice, requiring a shift from focusing solely on individual behavior to broader systemic constraints (28). For instance, structural environmental challenges such as air pollution significantly reduce life expectancy, an impact that can be moderated by institutional-level policies such as renewable energy adoption (29). This implies that the objective conditions for citizen participation in co-production are largely preconfigured by their socioeconomic status and environmental context.

Citizens’ co-production behaviors comprise two important aspects, which are also two significant objectives for government information disclosure, the dissemination of accurate information and the encouragement of citizens to comply with preventive behaviors (30). During the outbreak of a public health emergency, social unrest, lockdowns, and strict quarantines hinder information communication, making information exchange even more crucial (4). In this situation, government information disclosure can provide more credible information about public health crisis and facilitate citizens’ experience sharing, strengthening social support through their online networks, thereby fostering citizens’ co-production (31). Therefore, the first type of citizens’ co-production behavior is online engagement, search, and sharing of information which called online information concern behavior. In addition to information exchange, governments will announce a series of measures to control the spread of health crisis after the outbreak. For example, during the COVID-19 pandemic, people were encouraged to maintain social distancing. At this time, when the government discloses relevant information on public health crisis, including the number of confirmed cases and travel records, it can enhance citizens’ risk perception, leading them to voluntarily comply with the prescribed measures and promote co-production. Therefore, another important aspect of citizens’ co-production behavior is offline compliance with preventive behaviors and participation in volunteering for pandemic prevention and control which called offline compliance with preventive behaviors.

### 2.2 Signaling theory

Signaling theory, originally formulated by Spence (32), provides a robust analytical framework for explaining how organizations resolve information asymmetry by transmitting credible signals to external stakeholders (33). In the context of a public health crisis, a profound information gap exists between the government and the public, creating a state of high systemic uncertainty. We conceptualize government information disclosure as a critical institutional signal emitted by the signaler (the government) to reduce this asymmetry and foster social order (34). According to this theory, the efficacy of a signal is contingent upon its signal quality, which in our study is represented by two primary dimensions: timeliness and the level of detail (35).

The effectiveness of this signal is contingent not only upon its qualitative characteristics but also on the moderation of institutional dimensions. Research suggests that the policy-making environment dictates the efficacy of health policy integration across diverse sectors, which subsequently shapes the public’s reception of institutional signals (36). In the realm of digital public health governance, the synergy between technology and co-production is evident; for instance, digital subsidy schemes leverage ICT tools to enhance digital inclusion, thereby surmounting structural barriers to co-production (37). Furthermore, digital governance facilitates real-time interactions via platforms, significantly augmenting the efficiency of online information-seeking and mitigating cognitive barriers to information acquisition (30). The integration of digital intelligence allows for the personalized dissemination of institutional signals based on behavioral patterns, which bolsters citizens’ inclination toward online co-production (38). Beyond these digital facilitative mechanisms, the realization of actual co-production behavior is further predicated on the complex interplay between endogenous factors and external conditions. Offline policy compliance is primarily moderated by institutional trust and risk perception, which function as internal filters for external signals (39). Moreover, research on health outcomes, such as prematurity, demonstrates that outputs result from the dynamic interplay between endogenous biological factors and exogenous environmental stressors (40). Ultimately, government information disclosure can only effectively trigger co-production behavior when the institutional signal aligns with the bio-psycho-social state of the citizens.

High-quality signals are perceived as more credible because they require the signaler to invest significant administrative and cognitive resources, thereby serving as a proxy for governmental competence and reliability (33). Specifically, timeliness functions as a signal of institutional responsiveness, indicating the government’s monitoring capabilities and its commitment to public safety. In contrast, the level of detail signals administrative transparency and meticulousness. This signaling framework allows for a rigorous analysis of how the trade-offs in signaling strategies—speed versus granularity—influence the receiver’s cognitive processing and subsequent behavioral responses during emergencies. By interpreting these signals, citizens evaluate the severity of the crisis and the trustworthiness of the authorities, which directly determines their propensity to engage in co-production.

### 2.3 Information disclosure and citizens’ co-production

Government information disclosure not only contributes to improving the government’s image (41) and enhancing the level of government management (42, 43) but also increases the accuracy and rationality of public decision-making and behavior (38, 44, 45). During a public health emergency, society can fall into a state of chaos due to the severity and suddenness of the crisis. Normal communication channels between the government and citizens become disrupted. A scarcity of accurate and reliable information becomes a major challenge, hindering social stability and impeding citizens’ co-production (46). False information and rumors often emerge in times of crisis (4, 47, 48). For example, during the early stage of the COVID-19, the lack of timely and transparent information led to the spread of numerous rumors on social media (5). In times of emergency, citizens tend to become fearful and anxious (5), making it challenging to engage them effectively in co-production efforts. They may even tend to believe false information and take inappropriate actions, resulting in harmful consequences. Additionally, delayed information disclosure can negatively influence citizens’ evaluation of the government. For instance, the restrictions on information disclosure policy in Turkey were associated with a 26% decrease in citizens’ confidence in the accuracy of government-released information (49). During a public health emergency, effective information disclosure can effectively pacify public emotions, enhance crisis response capabilities, mitigate the adverse impacts of the crisis (45), and ultimately improve citizen co-production (2).

From the perspective of signaling theory, government information disclosure serves as a vital “institutional signal” in the process of citizen co-production. In environments characterized by extreme uncertainty, these signals act as a critical mechanism for reducing information asymmetry between the government and the public and for restoring social order. Government information disclosure plays a crucial role in citizens’ co-production. Public health crisis is typically widespread and affect entire nations (2). During such crises, rumors and various pieces of information spread rampantly throughout society (50). Citizens and communities heavily rely on government-provided information to enable their participation in crisis recovery (7). Government information disclosure promotes co-production in two main ways. First, when social order is disrupted and reliable information is in high demand (18), timely information disclosure by the government can reduce social uncertainty and public panic about the unknown (51), enabling rational cooperative behavior. Without such information, some initiatives from non-governmental organizations and well-intentioned citizens may unintentionally impede the recovery of the crisis. Second, as a significant information provider, the government can establish unified guidelines and action plans so as to enhance public disease prevention skills and behaviors, facilitate citizen learning during public crises, and strengthen their willingness to take unified action (5, 48). In summary, while the existing literature has established a robust foundation for understanding the fundamental role of institutional transparency in crisis management, most studies continue to treat information disclosure as a binary or static variable. Drawing on signaling theory, we argue that the efficacy of government communication depends not only on what is disclosed but, more crucially, on how it is delivered—specifically regarding its timeliness and specificity. A significant scholarly gap remains in explaining how these distinct signal characteristics differentially drive online information engagement versus offline compliance behaviors. By deconstructing co-production into its constituent dimensions, this study aims to uncover the underlying mechanisms through which differentiated disclosure strategies mitigate public uncertainty and foster collective resilience. This conceptual framework directly informs our subsequent hypotheses regarding the trade-offs between ‘speed’ and ‘detail’ in government signals.

The effectiveness of these signals is contingent upon two essential factors: the level of detail (19) and the timeliness of the disclosure (19, 20). Following the outbreak of a public health emergency, general information disclosure may lead to greater panic among citizens. In the case of the COVID-19, if only the current number of confirmed cases (e.g., 120 cases) is announced without providing further details, citizens may become more concerned about whether there are confirmed cases in their neighborhood. This can result in panic purchase and reluctance to leave homes.

Drawing upon signaling theory, detailed information disclosure is not merely an expression of transparency but serves as a core “competence signal.” Signals with a high level of detail, by providing specific travel records or granular pandemic data, significantly reduce “informational ambiguity” for the receiver. When signals contain sufficient actionable details, citizens perceive the government as possessing robust administrative control and professional rigor, thereby increasing their propensity to comply with policy guidance. This signal-quality-driven accuracy in risk perception serves as the central driver for triggering co-production behaviors. Therefore, the level of detail in information disclosure is an essential factor influencing citizen co-production behavior, leading to the following hypotheses:

H1a: The impact of information disclosure on citizens’ online information concern behavior is expected to be stronger when the disclosure is detailed rather than general.

H1b: The impact of information disclosure on citizens’ offline compliance with preventive behavior is expected to be stronger when the disclosure is detailed rather than general.

The timeliness of information disclosure is another important influencing factor, and generally, more timely information disclosure is believed to have a better impact on the public (9, 52). Furthermore, within the framework of signaling theory, the timeliness of disclosure is characterized as a “responsiveness signal.” In a rapidly evolving crisis, the value of information exhibits high temporal decay, making prompt signal emission essential for the government to fill information voids and project positive expectations of proactive, efficient crisis management. If the government delays information disclosure for a significant period after an emergency occurs, it may lead to citizens’ doubting the government’s capabilities, resulting in decreased trust and unwillingness to contribute to crisis management and social stability. This immediate signal feedback is vital as it maintains public trust in the institution and prevents interference from rumor signals, thus lowering the psychological barriers to citizen co-production. Moreover, in the absence of timely institutional signals, citizens may turn to internet influencers or rely on hearsay for information, but such information cannot guarantee accuracy and may contribute to the spread of rumors and confusion regarding the credibility of information (1). Conversely, non-timely signals are interpreted by receivers as a lack of administrative capacity or responsiveness, which inhibits cooperative tendencies. When the public believes in misinformation, their actions based on it may have adverse effects. Therefore, this study hypothesizes:

H2a: The impact of information disclosure on citizens’ online information concern behavior is expected to be stronger when the disclosure is timely rather than non-timely.

H2b: The impact of information disclosure on citizens’ offline compliance with preventive behavior is expected to be stronger when the disclosure is timely rather than non-timely.

Additionally, there may be an interaction between the timeliness and the level of detail in information disclosure. The theoretical foundation for this interaction is rooted in the inherent tension between speed and accuracy in crisis communication. During the outbreak of a public health emergency, government agencies often face severe human resource constraints and extensive pressure for cognitive guidance (2, 19, 49). Detailed information disclosure typically involves extensive data verification and cross-departmental coordination, which is inherently time-consuming and may lead to release delays. Conversely, prioritizing absolute timeliness often necessitates sacrificing information details in favor of broader messaging. This trade-off implies that the effectiveness of one dimension is contingent upon the level of the other; this is because, under different combinations of urgency and information detail, citizens’ cognitive processing and subsequent cooperative behaviors exhibit significant differences. In the aftermath of the pandemic outbreak, governments often need to choose between information disclosure strategies that are “detailed but not timely enough” and those that are “general but timely.” Identifying which type of information disclosure has a more significant impact on citizens’ co-production behavior is one of the central focuses of this research. Therefore, this paper hypothesizes that:

H3: There is a significant interaction between the timeliness and level of detail in information disclosure.

## 3 Methods

### 3.1 Study 1: Experiment research

#### 3.1.1 Experimental design and subjects

This study selected prevention policies in China during COVID-19 to validate hypotheses mentioned earlier. China was selected as the research background because COVID-19 initially emerged in Hubei Province, China, and subsequently spread to other regions of the country. Being the first and one of the most severely impacted nations, China provides an ideal setting for testing our hypothesis. In addition, the nature of China as a nation determines that the citizens have a stronger dependence on government, and the influence of non-governmental organizations is relatively weak. The government has a stronger binding and guiding role on citizens. Chinese government policies have a significant role in disseminating official information and providing guidance to the public. This factor further enhances the research’s suitability for verifying our hypothesis.

The hypothesis was validated through an experiment that adopted a 2 × 2 between-subjects experimental design. To ensure conceptual consistency between the experimental manipulation and the econometric analysis, we defined the core variables as shown in Table 1. Information Detail primarily refers to the degree of information specificity. Detailed disclosure is characterized by high specificity, incorporating exhaustive data such as precise travel trajectories and case backgrounds, whereas general disclosure provides only aggregate summaries. Information Timeliness is operationalized as the temporal interval between the occurrence of a public health event and its official announcement, categorized into timely and non-timely disclosure.

**Table 1:** Variable description.

Variable type	Variable name	Variable definitions
Dependent variable	Co-production	Y_1_: Online information concern behavior. Behaviors such as searching, disseminating, and sharing online information Y_2_: Offline compliance with preventive behaviors. Behaviors such as complying and publicizing pandemic preventive behaviors
Independent variable	Information disclosure detail level	Detailed information disclosure: the disclosed content includes specific data and specific cases General information disclosure: only basic data are disclosed
	Information disclosure timeliness	Timely information disclosure: disclosure made within 1 day. Non-timely information disclosure: disclosure made after 3 days or more.

The experimental simulation in this study was conducted within the context of government information disclosure in Tianjin, China, and the sample comprised residents who were affected by the 2022 local epidemic and had directly experienced its associated prevention and control measures; to ensure both practical feasibility and representative diversity, a multi-channel recruitment strategy was implemented, combining online and offline approaches: online invitations were randomly distributed via official community WeChat groups spanning all six administrative districts of Tianjin, leveraging the organic reach of social networks to engage a broad segment of the population, while offline recruitment involved research assistants approaching eligible volunteers in person at community centers and other public venues across the same districts, thereby ensuring comprehensive geographic and demographic coverage; this integrated approach effectively balanced key demographic variables—including age and gender—across the participant pool, enhancing the external validity and internal reliability of the experimental findings, with all participants providing voluntary informed consent, signing confidentiality agreements, and participating in offline sessions under protocols approved by the institutional ethics committee; following recruitment, a total of 165 individuals were enrolled, comprising 44.85% males and 55.15% females, and after rigorous manipulation checks, 158 valid responses were retained for subsequent analysis.

#### 3.1.2 Experiment task and process

The experimental participants were randomly divided into four distinct groups, which included the general disclosure group plus non-timely group, general disclosure group plus timely group, detailed disclosure group plus non-timely group, and detailed disclosure group plus timely group. Each participant was assigned to only one of these groups and received a unique set of experimental materials, which they were required to complete independently. After finishing the assigned task, participants were requested to return the materials immediately and then answer related questions. The experimental materials used in this study consisted of four separate sections. Within the task background section, subjects were informed that on January 8th, 2022, the Omicron variant of COVID-19 first emerged in China, leading to a new outbreak of the pandemic in Tianjin. Participants were then instructed to imagine being present in Tianjin and experiencing the outbreak firsthand. Following the outbreak, the Tianjin Municipal Government promptly carried out an investigation and disclosed related information about the pandemic through their official account, “Jinyun.” Disclosure information was divided into two parts: specific data and descriptions of cases’ travel records for the detailed disclosure group, and only basic data for the general disclosure group. A detailed summary of the specific information for both groups can be found in Supplementary Appendix C. Timely information disclosure emphasizes the importance of “updating information within 10 hours of an outbreak”. Conversely, non-timely information disclosure emphasizes “disclosure of information within 3 days of the outbreak”.

After reading the relevant materials, participants were asked to answer questions related to the dependent and control variables used in this study. The question regarding to the dependent variable Y_1_ was, “To what extent did you pay attention to information dissemination related to the Tianjin pandemic? (7-point scale, a higher number indicate higher levels of concern).” This question aimed to measure citizens’ online information concern behaviors in relation to co-production behaviors. The question regarding to the dependent variable Y_2_ was “To what extent were you willing to voluntarily comply with pandemic preventive behaviors, such as actively maintaining social distancing, participating in anti-epidemic volunteer services and so on? (7-point scale, a higher number indicate higher levels of compliance).” This question aimed to measure citizens’ offline compliance with preventive behavior in relation to co-production behaviors. Participants were then required to provide demographic information. Descriptive statistics for the variables can be found in Table 2.

**Table 2:** Descriptive statistics for variables.

Information disclosure detail level	Information disclosure timeliness			
	Y_1_		Y_2_	
	Timely	Non-timely	Timely	Non-timely
Detailed	6.49 (0.668) *N* = 43	5.63 (1.532) *N* = 38	6.42 (0.698) *N* = 43	5.45 (1.408) *N* = 38
General	6.33 (0.828) *N* = 36	6.41 (0.670) *N* = 41	5.33 (1.394) *N* = 36	5.34 (1.559) *N* = 41

Standard errors of the mean in parentheses.

#### 3.1.3 Manipulation check

To evaluate the effectiveness of manipulating two independent variables in the experiment, participants were required to answer questions related to the manipulation of disclosure content and disclosure timeliness. Specifically, they were asked, “What do you think is the level of detail included in the information disclosure? (General or Detailed)” and “Is the timeliness of the information disclosure timely or non-timely?” The results of the responses from 158 out of 165 residents who participated in the experiment indicate that the manipulation was successful.

#### 3.1.4 Repetition of experiment

To further validate the reliability of the experimental results, we conducted a round of repeated experiments. This round involved online random recruitment of participants, totaling 258 individuals. In this phase, we recognized that the previous measurement of online co-production behavior primarily focused on the level of information attention, which has certain limitations. Therefore, we introduced a measurement for the issue of information forwarding and sharing, represented as Y1 for “To what extent did you share and publicize information dissemination related to the Tianjin pandemic”. For measuring offline co-production behavior, we took into account the relatively stringent epidemic prevention requirements in China during the pandemic, such as mandatory mask-wearing when going outside. The question in Y21 is “To what extent are you willing to voluntarily comply with pandemic prevention behaviors, such as keeping a social distance, actively disinfecting, and volunteering to fight the pandemic? (on a 7-point scale, with higher numbers indicating higher levels of compliance)”. Behaviors in this category are mainly derived from citizens’ spontaneous behaviors. The question for Y22 is “To what extent are you willing to voluntarily comply with pandemic prevention behaviors, such as wearing a mask in public places, following home isolation requirements, etc.? (on a 7-point scale, with higher numbers indicating higher levels of compliance)”. This type of behavior is partly due to governmental requirements. The results of this experiment were consistent with those of the first round, thereby confirming the validity of Study 1. Detailed results are shown in Supplementary Appendices D,E.

### 3.2 Study 2: Regression analysis

Although Study 1 validated the significant impact of the level of information disclosure detail and timeliness on citizens’ co-production behavior through experimental methods, it did not determine the specific direction (positive or negative) of this impact. Furthermore, the design of Study 1 was rooted in the specific context of the Omicron outbreak in Tianjin, with a sample primarily composed of residents who personally experienced that period; moreover, the experimental method utilized involved certain sampling errors and subjective biases in questionnaire responses. Regarding measurement, Study 1 primarily focused on citizens’ behavioral intentions rather than objective actual behaviors, which presented certain limitations and subjective biases in reflecting data authenticity. Given these considerations, we further validate the research hypotheses through Study 2. Study 2 adopts regression analysis and investigates the information disclosure practices of all provinces and cities in China, which can provide further verification for the hypotheses.

The selected data for this regression analysis includes information disclosed by various provinces and cities in China between January 2020 and the end of February. Firstly, when COVID-19 first broke out in January 2020, China was the first country facing the pandemic with little prior experience to draw from. In comparison, subsequent countries had learned from China’s experience and adapted to deal with the pandemic. This discrepancy may have influenced citizen’s behaviors in cooperating with the government and accessing information from other sources. Secondly, provinces in China have varying degrees of autonomy in governance, resulting in varying degrees of information disclosure practices (2). This research context can provide valuable insights in better analyzing the relationship between government information disclosure and human behavior. Hence, the selection of data from different provinces will help facilitate a comprehensive study of the topic at hand. Therefore, this paper focuses on government information disclosure and human behavior in 293 prefecture-level cities in mainland China from January 16 to February 29, 2020- the most intensive period of COVID-19 spread (7).

### 3.3 Variable settings

#### 3.3.1 Information disclosure

Starting from January 20th, 2020, municipal Chinese governments at various levels have taken timely measures to release information to the public. The public information included the latest updates on the pandemic situation and the confirmed cases’ travel records. This information was primarily published on WeChat Official Accounts and Weibo. We referred to previous literature’s classifications to divide the information into general disclosure and detailed disclosure. The information on the number of daily confirmed cases is considered general information disclosure, whereas the information on commuting routes is classified as detailed information disclosure (5, 20). Additionally, the information was sorted into three categories based on its release time: within 12 h, 12–24 h, and more than 24 h, to represent the timeliness of information disclosure. We collected epidemic information released by various cities in China from January 16th to February 29th, 2020 (the outbreak period). This information was primarily published on WeChat Official Accounts and Weibo. The disclosed information includes the disclosure time, the number of confirmed cases, commuting routes of confirmed cases, etc.

#### 3.3.2 Co-production behavior

Traditional research methods relied on subjective indicators such as citizens’ cooperative willingness and attitudes (20). However, subjective attitudes and willingness may not always accurately measure citizens’ co-production behavior. In this study, we rely on real-time data that reflect the citizens’ actual behaviors, such as the number of individuals who consciously maintain social distance and wear masks. However, collecting accurate real-time data is challenging, and some actions might not represent voluntary behavior due to China’s strict prevention policies. Therefore, this paper considers the role of social networks in citizens’ co-production behavior. Previous studies on public crisis have used online behavioral indicators, such as the number of share and reading on WeChat, as well as web browsing data, to measure citizen behavior (7, 18). Therefore, this paper employed two main indicators to measure citizens’ co-production behavior. The first is the “sharing times” and “reading times” of government-disclosed information, which is measured online information concern behavior in co-production. It is used to gauge the government-citizen interaction and level of online contact. The second indicator is the frequency of citizen search queries on pandemic preventive behaviors, obtained from Baidu Index, the most popular search engine in mainland China (2, 7). We collected daily search data from prefecture-level cities manually, focusing on keywords “wearing masks” and “sterilize,” in order to gauge citizens’ offline compliance with preventive behavior in co-production. The index was calculated by assigning weights to the search frequencies of the keywords “wearing masks” and “sterilize” on both personal computers and mobile phones, with a higher value indicating more frequent and intensive search queries.

The selection of this indicator to measure citizens’ offline compliance with preventive behaviors was based on several significant reasons. Firstly, online searching shows how individuals efficiently seek and absorb information related to “wearing masks” and “sterilize.” It essentially reflects citizens’ adherence to preventive behaviors (7). A low volume of search queries indicates little attention being given to preventive measures. It is difficult to imagine that individuals would demonstrate high cooperation and compliance with preventive behaviors if they show little concern for “wearing masks” and “sterilize.” On the contrary, a high search query volume indicates that citizens have devoted more time and effort to search for relevant information, displaying increased awareness of preventive behaviors and potentially being better prepared to undertake necessary preventive actions (53). Previous studies have found that individuals who pay more attention to COVID-19 online are more likely to show higher levels of social engagement (7). This finding provides further evidence supporting the reliability and relevance of using search queries as an indicator for citizen co-production. Secondly, the Chinese government implements relatively strict control measures during public health crisis, such as city blockades. Some offline compliance with preventive behaviors is mandated by the government, online search queries regarding compliance with preventive behaviors during public health crisis provide a clearer picture of the voluntary nature of co-production (31).

#### 3.3.3 Variable control

To reduce the differences among various prefecture-level cities, this study employed corresponding control variables. *Per Capita* Gross Regional Product ( Pc_GRP ) is the extent of economic development for a city. Urban Resident Population ( ${\mathrm{Pop}}_{i}$ ) measures the resident population of a city. Local General Public Budget Revenue ( ${\text{Fiscal}}_{i}$ ) measures a city’s financial situation. Number of Beds of Hospitals ( $\text{Hospitals}\_{\text{Beds}}_{i}$ ) measures the medical resources of a city. Number of Employees Joining Basic Medical Care System ( ${\text{Employees}}_{i}$ ) measures the social security situation for a city. References for selection of control variables Huang et al. (2).

### 3.4 Models and research methods

In this study, we employed a negative binomial regression model to measure the impact of government disclosure on citizens’ co-production behavior. Negative binomial regression models are commonly used to evaluate interventions for infectious diseases like H1N1 and HIV (2, 54). This model is specifically chosen because our dependent variables are count-based and exhibit significant over-dispersion—where the variance substantially exceeds the mean. For instance, the standard deviation of online sharing is notably higher than its mean, making the negative binomial model more statistically robust than a standard Poisson regression. Regarding the independent variable Disclosure_Time, we utilized an ordinal coding of 1, 2, and 3 to represent disclosure within 12 h, 12–24 h, and more than 24 h, respectively. This ordinal approach is adopted to capture the diminishing marginal utility of information timeliness—often referred to as the golden window of crisis communication. It allows us to examine whether delays beyond specific thresholds lead to a linear or non-linear decline in citizen co-production, providing more actionable insights for government decision-making. By controlling for pre-existing conditions such as local economy, population, financial status, and medical resources, this model allows us to measure the effect of government information disclosure on citizens’ co-production behavior during the pandemic outbreak. The overall design of our model is shown in Equation (1):


$$\begin{array}{l} \ln[E(Y_{i,t+1})]=\beta_{0}+\beta_{1}\;{\text{Disclosure}}_{\text{Time}i,t}\\ +\beta_{2}\;{\text{Disclosure}}_{\text{Content}i,t}+\beta_{3}\;{\mathrm{Pop}}_{i,t}+\beta_{4}\;\mathrm{Pc}\_{\mathrm{GDP}}_{i,t}\\ +\beta_{5}\;\text{Hospitals}\_{\text{Beds}}_{i,t}+\beta_{6}\;{\text{Employees}}_{i,t}\\ +\beta_{7}\;{\text{Fiscal}}_{i,t}+\varepsilon_{i,t+1}\;\; \end{array}$$ (1)

Among them, the dependent variable Y is the co-production behavior of citizens, including the following four aspects. Y_1_ (Share) refers to the number of likes and retweets after the government releases the corresponding information disclosure content through WeChat or Weibo. Y_2_ (Readings) means the view times of citizens after the government releases the corresponding information disclosure content through WeChat or Weibo. Y_3_ (Search for “wearing masks”) is the search data for “wearing masks” in Baidu Index. Y_4_ (Search for “sterilize”) is the search data for “sterilize” in Baidu Index.

The independent variable $\text{Disclosure}\_{\text{Time}}_{i,t-1}$ refers to the time when the government discloses information, where the information disclosure within 12 h represents 1, the information disclosure within 12–24 h is 2, and the information disclosure more than 24 h is 3. The independent variable $\text{Disclosure}\_{\text{Contnt}}_{i,t-1}$ refers to the level of detail of the government’s information disclosure, the detailed disclosure represents 2, and the general disclosure 1.

Due to the different outbreak times of each city, some cities will not disclose any information when there is no pandemic spread in the city. Therefore, the researchers excluded the data that did not disclose information. Finally, A total of 5,800 data were collected from 293 cities in this study, whose descriptive statistics are shown in Table 3. We carried out the maximum-likelihood estimation for the negative binomial model. The result shows that the absolute value of the O value is greater than 1.96, and the *p*-value is less than 0.05, indicating that negative binomial regression is appropriate (Supplementary Appendices A,B).

**Table 3:** Analysis of variance.

Variables	Type III sum of squares (SS)	df	Mean squares (MS)	*F*	Sig.
Y_1_					
Information disclosure detail level	3.877	1	3.877	4.046	0.046
Information disclosure timeliness	5.911	1	5.911	6.170	0.014
Information disclosure detail level * information disclosure timeliness	8.650	1	8.650	9.029	0.003
Error	147.538	15	0.958	--	--
Y_2_					
Information disclosure detail level	13.946	1	13.946	8.290	0.005
Information disclosure timeliness	9.117	1	9.117	5.419	0.021
Information disclosure detail level * information disclosure timeliness	9.428	1	9.428	5.604	0.019
Error	259.079	15	1.682	--	--

## 4 Results

### 4.1 Study 1: Experiment results and analysis

#### 4.1.1 Analysis of variance (ANOVA)

To evaluate the validity of the hypothesis, the study 1 conducted ANOVA analyses (55). The results of the ANOVA are presented in Table 4. Firstly, concerning the dependent variable Y_1_, the study found significant main effects of information disclosure detail [F (1,154) = 4.046, *p* = 0.046 < 0.05], and disclosure timeliness [F (1,154) = 6.170, *p* = 0.014 < 0.05], as well as a significant interaction effect [F (1,154) = 9.029, *p* = 0.003 < 0.01]. Secondly, with respect to the dependent variable Y2, the study observed significant main effects of information disclosure detail level [F (1,154) = 8.290, *p* = 0.005 < 0.01], and information disclosure timeliness [F (1,154) = 5.419, *p* = 0.021 < 0.05], as well as a significant interaction effect [F (1,154) = 5.604, *p* = 0.019 < 0.05].

**Table 4:** Simple main effects analysis.

Variables	Type III sum of squares (SS)	df	Mean squares (MS)	*F*	Sig.
Y_1_					
Effect of detail level in disclosure when information is disclosed non-timely	12.093	1	12.093	12.622	0.001
Effect of detail level in disclosure when information is disclosed timely	0.471	1	0.471	0.492	0.484
Effect of timeliness in disclosure when information is disclosed detailed	14.809	1	14.809	15.457	<0.001
Effect of timeliness in disclosure when information is disclosed general	0.127	1	0.127	0.132	0.717
Y_2_					
Effect of detail level in disclosure when information is disclosed non-timely	0.221	1	0.221	0.131	0.717
Effect of detail level in disclosure when information is disclosed timely	23.079	1	23.079	13.719	<0.001
Effect of timeliness in disclosure when information is disclosed detailed	19.029	1	19.029	11.311	0.001
Effect of timeliness in disclosure when information is disclosed general	0.001	1	0.001	0.001	0.978

#### 4.1.2 Simple main effects analysis

For the dependent variable Y_1_ and Y_2_ with interaction, the study 1 conducted a simple effect analysis which is presented in Table 5. For variable Y_1_, first, in the case of non-timely information disclosure, the effect of the disclosure content is prominent [F (1,154) = 12.622, *p* = 0.001 < 0.01]. Specifically, general disclosure (*M* = 6.41) has a more significant influence on the online information concern behavior than detailed disclosure (*M* = 5.63). Second, in the case of detailed information disclosure, the timeliness of disclosure has a significant effect [F (1,154) = 15.457, *p* < 0.001], and timely disclosure (*M* = 6.49) has a greater impact on citizens’ online information concern behavior than non-timely disclosure (*M* = 5.63). Third, the effect of disclosure content is not significant in the case of timely information disclosure [F (1,154) = 0.492, *p* = 0.484 > 0.05]. Fourth, when the content of the disclosure is general, the timeliness of the disclosure has no significant effect [F (1,154) = 0.132, *p* = 0.717 > 0.05]. Fifth, a further comparison between detailed non-timely disclosure and general timely disclosure shows that the effect of “detailed but non-timely” disclosure (*M* = 5.63) is lower than that of “general but timely” disclosure (*M* = 6.33) in influencing citizens’ online information concern behavior. This finding suggests that if the purpose of the disclosure is to disseminate information and encourage people to pay attention to the information, it is more recommended to adopt a general but timely method.

**Table 5:** Descriptive statistics of main variables.

Variables	Mean	STD
Y_1_ (Share)	25116.846	30842.209
Y_2_ (Readings)	1119.177	296.724
Y_3_ (Search for “masks”)	468.905	539.934
Y_4_ (Search for “disinfectants”)	33.807	46.300
$\text{Disclosure}\_{\text{Time}}_{i,t-1}$	1.162	0.394
$\text{Disclosure}\_{\text{Contnt}}_{i,t-1}$	1.296	0.457
${\mathrm{Pop}}_{i}$	956.380	456.317
$\mathrm{Pc}\_{\mathrm{GRP}}_{i}$	9.798	3.635
$\text{Hospitals}\_{\text{Beds}}_{i}$	8.438	1.165
${\text{Employees}}_{i}$	818.716	2.793
${\text{Fiscal}}_{i}$	5.340	3.456

Regarding the variable Y_2_, first, in the case of timely information disclosure, the effect of the information disclosure detail level is significant [F (1,154) = 13.719, *p* < 0.001]. The results show that detailed disclosure (*M* = 6.42) has a greater influence on the offline compliance with preventive behaviors during the pandemic compared to general disclosure (*M* = 5.33). Second, in the case of detailed information disclosure, the timeliness of disclosure has a significant effect [F (1,154) = 11.311, *p* = 0.001 < 0.01], with timely disclosure (*M* = 6.42) having a higher impact on the offline compliance with preventive behaviors during the pandemic than non-timely disclosure (*M* = 5.45). Third, in the case of non-timely information disclosure, the effect of the disclosure content is not significant [F (1,154) = 0.131, *p* = 0.717 > 0.05], indicating that if the disclosure is not timely, detailed content or general content influence is not significant. Fourth, the effect of timeliness of disclosure is not significant when general information is disclosed [F (1,154) = 0.001, *p* = 0.978 > 0.05], indicating that if general information is disclosed, whether it is timely has little impact. Fifth, the effect of detailed non-timely disclosure (*M* = 5.45) is higher than that of general timely disclosure (*M* = 5.33). If disclosure is to comply with and publicize pandemic preventive behaviors in co-production behaviors, detailed but not timely methods are advised.

### 4.2 Study 2: Regression result and analysis

The negative binomial regression results were shown in Table 6, with (1) and (2) representing whether there is a control variable to reduce its impact. The results showed that for the dependent variable Y_1_, information disclosure timeliness has a significant negative effect (*β* = −0.730; *p* < 0.001), whereas information disclosure detail level has a significant positive effect (*β* = 0.977; *p* < 0.001). With the dependent variable Y_2_, information disclosure timeliness has a significant negative effect (*β* = −0.740; *p* < 0.001), and information disclosure detail level has a significant positive effect (*β* = 0.930; *p* < 0.001). For the dependent variable Y_3_, information disclosure timeliness has no significant effect (*β* = 0.057; *p* > 0.05), while information disclosure detail level has a significant positive effect (*β* = 0.112; *p* < 0.001). With the dependent variable Y_4_, information disclosure timeliness has a significant negative effect (*β* = −0.122; *p* < 0.001), and information disclosure detail level has a significant positive effect (*β* = 0.262; *p* < 0.001).

**Table 6:** Negative binomial regression results (regression coefficients, significance).

Variables	Y_1_ (Share)		Y_2_ (Readings)		Y_3_ (Search for “masks”)		Y_4_ (Search for “disinfectants”)	
	(1)	(2)	(1)	(2)	(1)	(2)	(1)	(2)
$\text{Disclosure}\_{\text{Time}}_{i,t-1}$	−0.730^***^ (0.035)		−0.740^***^ (0.034)		0.057 (0.033)		−0.122^***^ (0.034)	
$\text{Disclosure}\_{\text{Contnt}}_{i,t-1}$	0.977^***^ (0.030)		0.930^***^ (0.029)		0.112^***^ (0.029)		0.262^***^ (0.030)	
${\mathrm{Pop}}_{i}$	0.000^***^ (0.000)	0.000^***^ (0.000)	0.000^***^ (0.000)	0.000^***^ (0.000)	0.001^***^ (0.000)	0.001^***^ (0.000)	0.002^***^ (0.000)	0.002^***^ (0.000)
$\mathrm{Pc}\_{\mathrm{GRP}}_{i}$	0.073^***^ (0.004)	0.095^***^ (0.004)	0.054^***^ (0.004)	0.065^***^ (0.004)	0.044^***^ (0.004)	0.044^***^ (0.004)	0.054^***^ (0.004)	0.057^***^ (0.004)
$\text{Hospitals}\_{\text{Beds}}_{i}$	−2.048^***^ (0.058)	−1.693^***^ (0.058)	−1.266^***^ (0.058)	−0.914^***^ (0.057)	−0.068 (0.057)	−0.044 (0.057)	−0.511^***^ (0.058)	−0.436^***^ (0.059)
${\text{Employees}}_{i}$	−1.262^***^ (0.036)	−1.043^***^ (0.035)	−0.779^***^ (0.035)	−0.562^***^ (0.035)	−0.041 (0.035)	−0.026 (0.035)	−0.314^***^ (0.036)	−0.268^***^ (0.036)
${\text{Fiscal}}_{i}$	0.414^***^ (0.012)	0.343^***^ (0.012)	0.260^***^ (0.012)	0.189^***^ (0.012)	0.016 (0.011)	0.011 (0.012)	0.104^***^ (0.012)	0.089^***^

(1) All models; (2) Control variable models.

After conducting regression analysis and experimental tests, our findings show that: (1) Information disclosure timeliness has a significant negative impact on online information concern behavior in co-production, and H_2a_ is supported. Additionally, information disclosure detail level has a significant positive impact on behavior, H_1a_ is supported. (2) There is a significant interaction between information disclosure timeliness and information disclosure detail level with regards to online information concern behavior and H_3_ is supported. In the case of non-timely information disclosure, general disclosure has a more significant impact on behavior. In contrast, in the case of detailed information disclosure, the impact of timely disclosure is more significant than non-timely disclosure. (3) “General but timely” disclosure has a more significant impact on online information concern behavior than “detailed but non-timely” disclosure. (4) Regarding offline compliance with preventive behavior in co-production, information disclosure timeliness has a significant negative impact, and H_2b_ is supported. However, when the regression analysis only verifies the dependent variable search for “wearing masks,” the impact of information disclosure timeliness is not significant. This indicates that the impact of timeliness on some behaviors of pandemic prevention compliance is not evident and requires further exploration. Information disclosure detail level has a significant positive impact, and H_1b_ is supported. (5) There is a notable interaction between information disclosure timeliness and information disclosure detail level when studying offline compliance with preventive behavior, and H_3_ is supported. The impact of information disclosure on behavior differs depending on the timing and level of detail. Specifically, we found that for timely information disclosure, detailed disclosure has a more significant impact on behavior, whereas for detailed information disclosure, the impact of timely disclosure is more significant than non-timely disclosure. (6) “Detailed but non-timely” disclosure has a more significant impact on offline compliance with preventive behavior than “general but timely” disclosure.

## 5 Discussion and conclusion

### 5.1 Key findings

In conclusion, the research findings presented in this paper revealed the following noteworthy outcomes. By situating these results within the broader discourse of public administration, we offer a more nuanced perspective that departs from the traditional transparency paradigm, which often posits that “more information is always better.” Unlike existing research, our results indicate that the effectiveness of informational detail is fundamentally contingent upon its timeliness, particularly regarding online information concern behaviors.

Firstly, the effective management of public health crisis necessitates timely and comprehensive dissemination of information in order to encourage citizen’ involvement in co-production behavior. This observation is consistent with signaling theory, suggesting that signal quality is determined not only by its content but also by the temporal context of its transmission.

Secondly, the impact of information disclosure varies depending on the type of co-production behavior exhibited by citizens. For online information concern behavior, detailed and timely information disclosure is the most effective policy. However, due to limitations in government human resources and time, achieving detailed and timely information disclosure may be challenging. In such cases, a “general but timely” disclosure policy may be adopted. Our findings highlight that under such resource constraints, governments must adopt differentiated signaling strategies to guide different categories of behavior: specifically, prioritizing a “general but timely” policy for online information engagement, while favoring a “detailed but non-timely” approach to effectively promote offline compliance with preventive measures. This suggests that the strategic alignment between signal quality and behavioral objectives is a critical factor in successful crisis management.

### 5.2 Theoretical implications

This study contributes to the literature in three primary ways: First, it departs from traditional static classification paradigms of co-production by deconstructing the behavior into online information engagement and offline preventive compliance. By integrating signaling theory, this framework redefines government information disclosure as a critical institutional signal used to mitigate information asymmetry during public health crises. This conceptual shift allows the study to move beyond a binary disclosure vs. non-disclosure perspective and instead explore how these administrative signals interact with citizens’ cognitive processing across different participation dimensions. Specifically, it uncovers the nuanced mechanisms through which differentiated disclosure strategies reduce public uncertainty and foster collective resilience.

Second, the study develops a comprehensive analytical approach that bridges objective and experimental data. At the macro level, regression analysis of over 5,800 data points from 293 cities reveals broader behavioral patterns, while micro-level behavioral experiments with 158 citizens capture individual decision-making dynamics. This sequential experimental-observational mixed-methods design provides robust empirical evidence for the entire signaling chain-from the emission of signal characteristics to individual psychological and behavioral responses. Such an approach significantly enhances the reliability of the findings and effectively mitigates the effect inflation often found in studies relying solely on self-reported data.

Third, from the perspectives of behavioral public management and signaling theory, this research reveals a matching mechanism between disclosure formats and co-production types. By constructing a 2 × 2 policy matrix of timeliness and specificity, this study identifies timeliness as a responsiveness signal that signals administrative efficiency, and specificity as a competence signal that conveys professional rigor. Our findings suggest that online engagement is more sensitive to responsiveness signals, whereas offline compliance is primarily driven by the concrete details in competence signals which serve to significantly reduce informational ambiguity. Consequently, the study proposes differentiated disclosure strategies, such as “timely but general” versus “detailed but delayed”, providing a rigorous theoretical foundation for governments to navigate the trade-offs between signal speed and detail under resource constraints.

### 5.3 Practical implications

In terms of practical significance, we primarily proposes effective government information disclosure strategies and recommendations. Firstly, the results have shown that information disclosure significantly impacts citizens’ co-production behavior (7). However, the effect of information disclosure varies for different co-production behaviors. When the purpose of government information disclosure is to encourage the public to pay more attention and spread accurate information related to the pandemic, the best approach is to disclose information that is “detailed and timely.” However, if there is a lack of time and human resources, it is recommended that the government adopt a “general but timely” form of information disclosure to release information promptly. Even if the information is not yet comprehensive, the timely information can quickly allow the correct information disclosed by the government to have a more significant impact on public opinion (1). The incompleteness of information will draw people’s continuous attention, driving them to seek out more information.

Secondly, it is essential to acknowledge that resource constraints and time limitations within the government make it challenging to balance timely and detailed information disclosure simultaneously (49). Sometimes the government is unable to adopt a “detailed and timely” form of information disclosure due to a lack of time and human resources. In this situation, studies have revealed that when the aim of government information disclosure is to encourage citizens to voluntarily follow pandemic prevention policies, it is recommended to adopt a “detailed but not timely” approach to information disclosure. This is because the level of detail in the information disclosure is more significant to citizens. By providing detailed information on the actual situation of the pandemic, the severity of the outbreak, and the travel records of confirmed cases, citizens can have a personal understanding of the gravity of the pandemic and be more likely to comply with prevention policies.

Thirdly, our study’s findings suggest that the government’s information disclosure policy is crucial in promoting citizens’ co-production behavior during public health crisis. However, it is important for the government to adjust its information disclosure policy to cater to different purposes, especially in the early stages of an outbreak when timely information is crucial for controlling rumors and emphasizing the role of official information (2). During the critical phase of the pandemic, the government should focus on promoting citizens’ compliance with prevention measures and encourage voluntary services (7). These two scenarios require targeted measures and should be studied separately. In accordance with recommendations one and two, different information disclosure policies should be implemented at different time points to promote specific types of co-production behaviors. This approach will ensure that information disclosure policies are tailored to the unique local conditions and characteristics of the population and implemented diligently.

### 5.4 Research limitations and future study

During times of public health crisis, the dissemination of information by the government can significantly influence citizens’ contribution to the collective efforts. This study specifically aims to analyze the interplay between different forms of information disclosure and citizens’ co-production behavior. We have identified that the interplay between the timeliness and level of detail in information disclosure has varying impacts on different aspects of citizens’ co-production behavior. Specifically, timely but general information disclosure tends to have a larger impact on online information concern behavior, while detailed but non-timely information disclosure is more effective in promoting offline compliance with preventive behavior. Our finding can offer practical recommendations for authorities to enhance information disclosure policies during emergency situations, specifically regarding public health crisis, and consequently improve the quality of government crisis management. In addition, this paper also contributes theoretically. The research extends the definition of citizen co-production behavior, categorizing it into online and offline behaviors, and investigates the influencing factors of each behavior separately. Furthermore, this paper explores specific forms of information disclosure and their relationship to co-productive behavior, enriching the model of information disclosure research.

However, this study is subject to several limitations. First, the classification of co-production behavior is restricted to online and offline categories, which may not capture the full complexity of citizen participation. Second, the sample is limited to residents of Tianjin, China, and the research may be influenced by certain methodological biases such as selection and response bias, where participants’ feedback might be affected by social desirability. Third, the use of online search indices as a proxy for offline behavior has inherent indirectness compared to actual observed actions. Furthermore, the findings are deeply rooted in China’s specific social governance system and a cultural context characterized by high public reliance on the government. This “state-centric” orientation potentially constrains the direct generalizability and external validity of the results to diverse institutional and political environments, such as liberal democracies.

In future studies, the research context should be expanded by applying the proposed analytical frameworks to countries with different political systems and conducting cross-national comparative studies to critically evaluate how institutional and cultural backgrounds influence co-production. To enhance external validity, data should be collected from larger and more diverse samples across various regions. Methodologically, future research should seek opportunities to conduct extensive field experiments during public health emergencies to measure actual participation more accurately than proxy metrics. Furthermore, it is essential to incorporate potential mediating mechanisms—such as risk perception, institutional trust, and cognitive load—to more comprehensively uncover the psychological pathways through which different disclosure forms shape co-production. Additionally, future research could explore the evolving role of digital technologies and artificial intelligence in public communication. Finally, extending the research conclusions to other types of crises, such as economic or climate crises, will further enhance the practical significance and universal applicability of the findings.

## Data Availability

The datasets presented in this study can be found in online repositories. The names of the repository/repositories and accession number(s) can be found in the article/Supplementary material.
