# Supplementary material for: Timely or detailed? The impact of information disclosure on citizen co-production
Source: Front Public Health. 2026 Jun 1;14:1774267. doi: 10.3389/fpubh.2026.1774267 (PMC13267687; doi:10.3389/fpubh.2026.1774267)
Supplement: Supplementary file 1 [file Table_1.docx]

**Appendix A: Negative binomial regression results (Z-values)**

| Variables | Y_1_ (Share) | | Y_2_(Readings) | | Y_3_(Search for " wearing masks ") | | Y_4_(Search for "sterilize") | |
| --- | --- | --- | --- | --- | --- | --- | --- | --- |
|  | （1） | （2） | （1） | （2） | （1） | （2） | （1） | （2） |
| ${Disclosure\_Time}_{i,t-1}$ | -21.075 |  | -21.684 |  | 1.728 |  | -3.579 |  |
| ${Disclosure\_Contnt}_{i,t-1}$ | 32.987 |  | 31.616 |  | 3.850 |  | 8.794 |  |
| $\mathrm{Pop}_{i}$ | 14.828 | 15.138 | 10.856 | 11.524 | 39.884 | 40.126 | 55.457 | 56.052 |
| ${Pc\_GDP}_{i}$ | 17.810 | 23.476 | 13.367 | 16.103 | 11.024 | 10.934 | 13.308 | 13.768 |
| ${Hospitals\_Beds}_{i}$ | -35.253 | -29.414 | -22.008 | -16.001 | -1.198 | -0.771 | -8.776 | -7.418 |
| $\mathrm{Employees}_{i}$ | -35.239 | -29.398 | -21.969 | -15.960 | -1.178 | -0.750 | -8.769 | -7.411 |
| $\mathrm{Fiscal}_{i}$ | 35.387 | 29.560 | 22.377 | 16.385 | 1.392 | 0.972 | 8.844 | 7.491 |

**Appendix B: Likelihood ratio test for negative binomial regression model (P-value, N-value)**

| Variables | Y_1_ (Share) | | Y_2_(Readings) | | Y_3_(Search for " wearing masks ") | | Y_4_(Search for "sterilize") | |
| --- | --- | --- | --- | --- | --- | --- | --- | --- |
|  | （1） | （2） | （1） | （2） | （1） | （2） | （1） | （2） |
| P | 0.000 | 0.000 | 0.000 | 0.000 | 0.000 | 0.000 | 0.000 | 0.000 |
| N | 5702 | 5702 | 5705 | 5705 | 5780 | 5780 | 5780 | 5780 |
| Test for overdispersion in O-value. | 39411.175（0.000） | 39451.990（0.000） | 2022140.669（0.000） | 2025868.603（0.000） | 33521.721（0.000） | 33204.283（0.000） | 3342.627（0.000） | 3338.550（0.000） |
| Note: If the absolute value of the O value is greater than 1.96 and the p-value is less than 0.05, it indicates that the data is highly scattered, and negative binomial regression is more appropriate to use in this case. | | | | | | | | |

**Appendix C**

**Detailed Information Disclosure**

On January 8th, 2022, a new outbreak of the pandemic occurred in Tianjin. If you were in Tianjin at that time and experienced the outbreak, the Tianjin Municipal Government promptly investigated the situation and disclosed relevant pandemic information through the official account ‘Jinyun’ The description below shows the specific content of information disclosure after the outbreak on January 8th. Please read it carefully and provide detailed disclosure：

**[Authoritative Release]** Report on the Status of COVID-19 Infections on January 8th:

The Tianjin Municipal Epidemic Prevention and Control Headquarters announced the latest news that on January 8th, 2022, the Omicron variant of COVID-19 spread in Tianjin, causing a new outbreak of the pandemic. From 0:00 to 24:00 on the 8th, 10 new locally transmitted confirmed cases were reported. The details are as follows:

**Case 1** is a 29-year-old female, not under quarantine, working in a childcare agency and living in Huayuan New City, Nankai District. Due to work needs, she often resides in Xinzhuang Town, Jinnan District. The patient did not leave Tianjin for the 14 days before the onset of symptoms. On the early morning of January 6th, the patient experienced chills, cough, and runny nose, and took self-medication when the highest self-monitored temperature was 36.9°C. On the 7th, the patient went to Jinnan Hospital for self-testing of nucleic acid and was confirmed positive for COVID-19 on the morning of the 8th. She was then transported to Haihe Hospital for treatment by ambulance. After a comprehensive analysis by the municipal expert team, the patient was diagnosed as a confirmed case of local COVID-19 (mild).

**Case 2** is a 10-year-old female student at Xian Shui Gu No. 7 Primary School, residing in Xianshui Gu Town, Jinnan District along with her parents. None of them had left Tianjin for the 14 days prior to the onset of symptoms. On January 7th, the patient and her family self-drove to Jinnan Hospital Fever Clinic for diagnosis. On the morning of the 8th, the patient was confirmed positive for COVID-19 via nucleic acid testing and subsequently was transported to Haihe Hospital for treatment by ambulance. After a comprehensive analysis by the municipal expert team, the patient was diagnosed as a confirmed case of local COVID-19 (mild).

**Case 3** is a 10-year-old male student at Beizhakou Town, Jinnan District, Tianjin, and a classmate of Case 2, with close contact with her. On January 8th, the patient was tested positive for COVID-19 via throat swab sample testing by the Jinnan District Disease Prevention and Control Center.

**Cases 4-5** are students at Xinzhuang Middle School, residing in Xinzhuang Town, Jinnan District, Tianjin, and are classmates in the same after-school program as Case 1, having close contact with him. On January 8th, they were tested positive for COVID-19 via throat swab sample testing by the Jinnan District Disease Prevention and Control Center.

**Cases 6-10** are students at Gaozhuangzi Elementary School, residing in Xinzhuang Town, Jinnan District, Tianjin, and are classmates in the same after-school program as Case 1, having close contact with him. On January 8th, they were tested positive for COVID-19 via throat swab sample testing by the Jinnan District Disease Prevention and Control Center.

The recent activity tracks of the two newly confirmed cases who were not under quarantine are as follows:

**Newly confirmed case 1:** January 2nd: Subway Line 6 Nancuiping Station (7:16) - Line 5 Tumor Hospital - Line 1 Xiawafang Station - Gaozhuangzi (8:30) - Bus Route 202 (8:34); January 3rd: Line 1 Gaozhuangzi Station (21:31) - Xiawafang Station - Line 5 Tumor Hospital - Line 6 Nancuiping Station (22:34); January 4th: Subway Line 6 Nancuiping Station (14:41) - Line 5 Tumor Hospital - Line 1 Xiawafang Station (15:13) - Gaozhuangzi (15:42); January 5th: Meihui Supermarket (7:11); January 6th: Ruicheng Drugstore (Xinzhuang Branch); January 7th: Subway Line 6 Nancuiping Station (13:00) - Xianshuigouxi Station (14:19).

**Newly confirmed case 2:** January 2nd: New Hua City Square Underground Supermarket in Jinnan District (11:14-11:58) - Youpinhui Glasses Shop (Huian Garden Branch) (12:00); January 3rd: Xianshuigu Market (10:12-11:27) - Youpinhui Glasses Shop (Huian Garden Branch) (12:30) - Xiaoliangkou Potato Noodles (Theater West Community Shop) (13:20); January 4th-7th: Xian Shui Gu No. 7 Primary School (7:20-17:10).

**General Information Disclosure**

On January 8th, 2022, a new outbreak of the pandemic occurred in Tianjin. If you were in Tianjin at that time and experienced the outbreak, the Tianjin Municipal Government promptly investigated the situation and disclosed relevant pandemic information through the official account ‘Jinyun’ The description below shows the specific content of information disclosure after the outbreak on January 8th. Please read it carefully and provide detailed disclosure：

**[Authoritative Release]** Epidemic Infection Situation Report on January 8th

The Tianjin Epidemic Prevention and Control Command Center has issued the latest message that on January 8, 2022, the Omicron variant of the new coronavirus had spread in Tianjin, sparking a new outbreak of the epidemic. From 00:00 to 24:00 on the 8th, there were 10 new local cases of COVID-19 infections that tested positive for nucleic acid. The specific situation of the cases and their activity trajectories are still under investigation, and further detailed information will be released later.

**Appendix D. *Repetition of Experiment* Descriptive statistics for variables**

| Information Disclosure Detail Level | Information Disclosure Timeliness | | | |  |  |
| --- | --- | --- | --- | --- | --- | --- |
|  | **Y_1:_** | | **Y_21_** | | **Y_22_** | |
|  | Timely | Non-timely | Timely | Non-timely | Timely | Non-timely |
| Detailed | 6.00  （0.79）  N=78 | 5.97  （0.97）  N=67 | 6.63  （0.63）  N=78 | 6.62  （0.55）  N=67 | 6.50  （0.64）  N=78 | 6.33  （0.81）  N=67 |
| General | 5.74  （1.01）  N=57 | 5.17  （1.37）  N=60 | 6.62  （0.62）  N=57 | 5.97  （1.13）  N=60 | 6.23  （0.87）  N=57 | 5.58  （1.23）  N=60 |

Note: standard errors of the mean in parentheses.

**Appendix E. *Repetition of Experiment* Analysis of Variance**

|  | Type III Sum of Squares（SS） | df | | Mean Squars(MS) | F | Sig. |
| --- | --- | --- | --- | --- | --- | --- |
| **Y_1_** | | | | | | |
| Information Disclosure Detail Level | 18.363 | 1 | 18.363 | | 17.084 | 0.000** |
| Information Disclosure Timeliness | 5.811 | 1 | 5.811 | | 5.406 | 0.021* |
| Information Disclosure Detail Level * Information Disclosure Timeliness | 4.712 | 1 | 4.712 | | 4.384 | 0.037* |
| Error | 277.326 | 258 | 1.075 | |  |  |
| **Y_21_** | | | | | | |
| Information Disclosure Detail Level | 6.963 | 1 | | 6.963 | 12.214 | 0.001** |
| Information Disclosure Timeliness | 7.165 | 1 | | 7.165 | 12.567 | 0.000** |
| Information Disclosure Detail Level * Information Disclosure Timeliness | 7.107 | 1 | | 7.107 | 12.466 | 0.000** |
| Error | 147.086 | 258 | | 0.570 |  |  |
| **Y_22_** | | | | | | |
| Information Disclosure Detail Level | 16.692 | 1 | | 16.692 | 21.019 | 0.000** |
| Information Disclosure Timeliness | 10.757 | 1 | | 10.757 | 13.545 | 0.000** |
| Information Disclosure Detail Level * Information Disclosure Timeliness | 3.613 | 1 | | 3.613 | 4.549 | 0.034* |
| Error | 204.895 | 258 | | 0.794 |  |  |
